# Conceptualizing Interprofessional Digital Communication and Collaboration in Health Care: Protocol for a Scoping Review

**DOI:** 10.2196/45179

**Published:** 2023-06-26

**Authors:** Kim Nordmann, Stefanie Sauter, Patricia Möbius-Lerch, Marie-Christin Redlich, Michael Schaller, Florian Fischer

**Affiliations:** 1 Bavarian Research Center for Digital Health and Social Care Kempten Germany

**Keywords:** digital technologies, interdisciplinary communication, intersectoral collaboration, nurse, physician, collaboration, interdisciplinary, scoping review, communication, interprofessional

## Abstract

**Background:**

Effective communication and collaboration among health professionals are essential prerequisites for patient-centered care. However, interprofessional teams require suitable structures and tools to efficiently use their professional competencies in the service of high-quality care appropriate to the patient’s life situation. In this context, digital tools potentially enhance interprofessional communication and collaboration and lead to an organizationally, socially, and ecologically sustainable health care system. However, there is a lack of studies systematically assessing the critical factors for successfully implementing tools for digitally supported interprofessional communication and collaboration in the health care setting. Furthermore, an operationalization of this concept is missing.

**Objective:**

The aim of the proposed scoping review is to (1) identify factors influencing the development, implementation, and adoption processes of digital tools for interprofessional communication in the health care sector and (2) analyze and synthesize the (implicit) definition, dimensions, and concepts of digitally supported communication and collaboration among health care professionals in the health care setting. Studies focusing on digital communication and collaboration practices among health care professionals, including medical doctors and qualified medical assistants, in any health care setting will be included in this review.

**Methods:**

To address these objectives, an in-depth analysis of heterogeneous studies is needed, which is best achieved through a scoping review. Within this proposed scoping review, which adheres to the Joanna Briggs Institute methodology, 5 databases (SCOPUS, CINAHL, PubMed, Embase, and PsycInfo) will be searched for studies assessing digital communication and collaboration among various health care professionals in different health care settings. Studies focusing on health care providers or patient interaction through digital tools and non–peer-reviewed studies will be excluded.

**Results:**

Key characteristics of the studies included will be summarized through descriptive analysis, using diagrams and tables. We will synthesize and map the data and conduct a qualitative in-depth thematic analysis of definitions and dimensions of interprofessional digital communication and collaboration among health care and nursing professionals.

**Conclusions:**

Results from this scoping review may help in establishing digitally supported collaborations between various stakeholders in the health care setting and successfully implementing new forms of interprofessional communication and collaboration. This could facilitate the transition to better coordinated care and encourage the development of digital frameworks.

**International Registered Report Identifier (IRRID):**

PRR1-10.2196/45179

## Introduction

### Overview

Efficient communication and collaboration among health care professionals are essential prerequisites for high-quality patient-centered care [1]. Among other things, this reduces readmission rates, improves patient health outcomes, increases time efficiency, partially improves clinical processes, and reduces health care costs [2-4].

Interaction among health care professionals can be denominated as inter-, multi- or transprofessional. Although the definition of each term is not uniform, they decipher different degrees of collaboration between professions [5]. In this review, we will use the term “interprofessional communication and collaboration” (ICC) to refer to any type of interprofessional interaction among health care professionals, from pure information exchange to collaborative working.

Key enablers for ICC are trust and respect, shared visions, as well as respectful and constant communication [6]. Barriers to ICC include a lack of funding and inadequate reimbursement, poor definitions of roles and responsibilities, insufficient training, a lack of time, hierarchical differences, and divergent communication styles [7-9]. Communication styles can be classified as either formally structured, such as team meetings or clinical rounds, or informal and opportunistic, such as hallway consultations [10].

Within our scoping review, we focus on 1 specific part of ICC, which is characterized by the use of digital tools to support its effectiveness [9,11]. Research shows that digitally supported interprofessional communication and collaboration (DICC) lead to better division of tasks; improve the definition of responsibilities; increase the accessibility, efficiency, and safety of clinical information transfer; and partly replace time- and resource-consuming face-to-face multidisciplinary meetings [2,12-15]. Despite the benefits, implementation of DICC is complex and challenging, with studies assessing critical factors for the successful implementation and use of DICC being scarce [16].

Although various definitions and conceptualizations of DICC are available in the literature, they largely neglect the technical dimension [16,17]. Greenhalgh et al [16] proposed the Nonadoption, Abandonment, Scale-up, Spread, and Sustainability (NASSS) framework, which helps predict and evaluate the success of a technology-supported health or social care program. Successful implementation of DICC tools depends on the material and technical features, the knowledge generated, the support and knowledge needed to use the tools, as well as their substitutability and sustainability. A definition of DICC that includes these dimensions may guide research activities and support implementation projects, improving the probability of success for DICC. Therefore, we aim to conduct a scoping review on the (implicit) definition and dimensions of DICC across health care settings and its driving factors.

A preliminary search of PubMed returned some systematic reviews on facilitators of and barriers to ICC [6,8,11,18] as well as a handful of scoping reviews on DICC in the primary health care system and for specific disease groups [19-24].

However, no current or ongoing PROSPERO-registered systematic or scoping review on our research aim was identified in previous primary and review studies.

### Aim and Review Questions

This review aims to (1) identify the factors that influence the development, implementation, and adoption processes of DICC and (2) elicit, analyze, and synthesize the (implicit) definition, dimensions, and concepts of DICC among health care professionals.

Subquestions driving the analysis include the following:

- How can DICC be operationalized?
- How do DICC complement or interfere with existing ICC practices and pathways?
- What barriers and solutions exist in the use of DICC?
- How does the health care setting (eg, primary care or secondary care) impact key concepts of DICC practices?

## Methods

### Search Strategy

To be able to conduct an in-depth analysis of the literature, we will draw upon a broad range of heterogeneous types of primary research, which can best be obtained through a scoping review. To standardize the approach, we will adhere to the Joanna Briggs Institute methodology [25].

In accordance with the methodology, we adopted a 3-step approach for including articles. To start with, an initial screening of titles, abstracts, and index terms of relevant articles in PubMed was used to identify possible keywords and refine our final search strategy (Table 1). Specific instant messenger apps, such as WhatsApp and WeChat, were included in the search if they were demonstrably the most popular messenger apps in at least one country and were specific to the search, meaning we discarded Line and imo due to their ambiguity [26]. Table S1 (Multimedia Appendix 1) details the hits per search string in PubMed. In a second step, we adapted the search strategy to each database (ie, CINAHL, PubMed, Embase, PsycInfo, and Scopus; Table S2 in Multimedia Appendix 1). Search strings were tested across all databases for conclusiveness. Our third step will be to manually screen all references of the included full texts for additional studies according to the snowball technique described by Greenhalgh et al [27]. Due to the rapid development of digital technologies, we will limit the studies included to those conducted from 2012 onward and include studies in English, French, German, Portuguese, and Spanish.

**Table 1:** Search strategy on MEDLINE via PubMed and SCOPUS.

Participant, concept, and context scheme	#	Search string	Hits on PubMed (December 11, 2022)	Hits on SCOPUS (December 11, 2022)
Communication and collaboration among different health care provider groups	1	trans-disciplin*^a^ OR transdisciplin*^a^ OR cross-disciplinar*^a^ OR crossdisciplinar*^a^ OR inter-disciplin*^a^ OR interdisciplin*^a^ OR multi-disciplin*^a^ OR multidisciplin*^a^ OR multi-profession*^a^ OR multiprofession*^a^ OR inter-profession*^a^ OR interprofession*^a^	185,543	392,636
	2	“knowledge transfer”^a^ OR information*^a^ OR Health Information Exchange^b^ OR cooperat*^a^ OR co-operat*^a^ OR collaborat*^a^ OR communicat*^a^	2,089,524	7,334,822
	3	“integrated care”^a^ OR Intersectoral Collaboration^b^ OR Interdisciplinary Communication^b^	26,343	33,558
	4	(# 1 AND #2) OR 3#	75,493	144,245
Digital tools	5	Health Information Systems^b^ OR Ambulatory Care Information Systems^b^ OR Information Technology^b^ OR technolog*^a^ OR socio-techni*^a^ OR sociotechni*^a^ OR mHealth^a^ OR eHealth^a^ OR digit*^a^ OR Electronic Health Records^b^ OR Public Health Informatics^b^ OR messag*^a^ OR messeng*^a^ OR app^a^ OR video*^a^ OR phone^a^ OR E-Mail*^a^ OR “E Mails”^a^ OR “E Mail”^a^ OR Email*^a^ OR “electronic mail”^a^ OR “electronic mails”^a^ OR “social media”^a^ OR WhatsApp^a^ OR Facebook^a^ OR Viber^a^ OR WeChat^a^ OR Telegram^a^ OR Kakotalk^a^	1,254,069	5,992,856
Health care setting	6	Health*^a^ OR hospital*^a^ OR care*^a^ OR caring^a^	5,596,504	7,922,757
Combined	7	#4 AND #5 AND #6	7261	11,056
Filters	8	#7 + English, French, Spanish, Portuguese, German, from 2012 onward	5694	8216

^a^Title or abstract.

^b^Medical Subject Headings term for PubMed search string and title, abstract, or keyword for SCOPUS search string.

### Eligibility Criteria

We defined several eligibility criteria based on formal issues, such as article type or language of publication, and issues related to the content of the manuscript, such as participants and context. These eligibility criteria are listed in Table 2 and described in detail below.

**Table 2:** Inclusion and exclusion criteria.

Feature	Inclusion criteria	Exclusion criteria
Article type	Any of the following published in a peer-reviewed journal: Primary research approach and study design Opinion pieces Guidelines Reviews Meta-analyses Meta-syntheses	Unpublished studies Non–peer-reviewed research papers Grey literature Conference abstracts Editorials Book chapters Records for which we are unable to obtain the full text Studies that focus on students of health care professions
Language	English French German Spanish Portuguese	All other languages
Participants	DICC^a^ among health care professionals, including medical doctors and nursing staff	Studies focusing on DICC between patient groups and health care practitioners Studies focusing on individuals in the same health care profession within the same setting
Concept	DICC involving either at least two different groups of health care professionals DICC among health care professionals working in similar roles but in different health care settings	All other DICC among health care professionals
Context	Any geographic and demographic health care setting	N/A^b^

^a^DICC: digitally supported interprofessional communication and collaboration.

^b^N/A: not applicable.

#### Participants

The scoping review will include studies that focus on DICC among health care professionals, including medical doctors and nursing staff. Studies focusing on DICC between patient groups and health care practitioners as well as among individuals of the same health care profession within the same setting will be excluded.

#### Concept

We will consider DICC either involving at least two different groups of health care professionals or among health care professionals working in similar roles but in different health care settings.

#### Context

The study will include any geographic and demographic health care setting, as understandings of DICC may vary among different groups and settings, such as primary and secondary health care systems. We will include sources, but not limit them to such, assessing the implementation, piloting, and adoption of new DICCs.

#### Types of Sources

We will consider any type of primary research approach and study design, opinion pieces, guidelines, reviews, meta-analyses, and meta-syntheses published in a peer-reviewed journal. We will not include any unpublished studies, non–peer-reviewed research papers, or grey literature. We will further exclude conference abstracts, editorials, and book chapters, as well as all records for which we are unable to obtain the full text. Furthermore, studies that focus on students of health care professions will be excluded.

### Study or Source of Evidence Selection

For screening, review, and data extraction, the web-based review manager Covidence (Veritas Health Innovation) will be used. We will upload all identified records and remove duplicates. Following the Joanna Briggs Institute methodology, all authors will independently screen the title and abstract of the first 50 records sorted by first author’s last name. Any discrepancies will be discussed to ensure a uniform understanding of the inclusion and exclusion criteria. All remaining records will be screened by 2 independent reviewers. Potentially relevant sources will be retrieved in full, and their details will be imported into Covidence. Two independent researchers will assess the full text against the inclusion criteria and note reasons for exclusion. Any discrepancies between reviewers’ decisions will be settled by an additional reviewer throughout all stages of the screening process. The results of the search and the study inclusion process will be reported in full in the final scoping review report and presented in a PRISMA-ScR (Preferred Reporting Items for Systematic Reviews and Meta-Analyses extension for Scoping Reviews) flow diagram [28].

### Data Extraction

Data will be extracted from all included records by 1 reviewer using MAXQDA (version 2022; CERBI GmbH). Specific categories for data extraction are predefined (Table 3) and include details about the study setting, participants, and key findings relevant to the review questions. The draft data extraction tool will be modified and revised as necessary during the process of data extraction. All modifications will be detailed in the scoping review.

We will extract data for sources that define DICC based on another reference (“secondary record”) into a second data extraction sheet—if the secondary record is available to the researcher. No further inclusion or exclusion criteria will be applied to secondary records.

**Table 3:** Categories for data extraction.

Category	Item
Source details	Journal Year Country of study setting Study type
Details or results extracted from source of evidence	Aim or purpose Definitions (communication, collaboration, and digital tool) Health care setting Participants NASSS^a^ framework: Domain 1: the condition or illness Domain 2: the technology Domain 3: the value proposition Domain 4: the adopter system Domain 5: the organization Domain 6: the wider context Domain 7: embedding and adaptation over time Other remarkable results

**^a^**NASSS: nonadoption, abandonment, scale-up, spread, and sustainability.

### Analysis

The NASSS framework will be used as the basis for data analysis [16,17]. It consists of 7 domains, as follows: condition or illness, technology, value proposition, adopter system (intended users), organization(s), and wider context (especially regulatory, legal, and policy issues). The seventh domain, which is crosscutting, considers how the domains interact and change over time [16]. Through the NASSS framework, researchers can identify and explain the complex manifestations in technology-supported change efforts. Using the dimensions of the NASSS framework as predefined analytical themes, we will analyze the extracted data following a thematic analysis approach [29]. If necessary, we will inductively establish new themes or subthemes alongside the data analysis process.

## Results

We will characterize the extracted data of all primary and secondary records by means of descriptive analysis, using diagrams and tables, accompanied by a narrative account of the findings, with a focus on the questions of this scoping review. A qualitative in-depth thematic analysis of definitions, dimensions, and concepts will be carried out, and data will be synthesized and mapped.

The scoping review was initiated in October 2022 with a protocol. Screening started in December 2022 and the analysis will be concluded in August 2023. We expect to submit the final scoping review manuscript by October 2023

## Discussion

### Contribution to Digital Health

The definitions and dimensions identified might have the potential to assist in the design, development, implementation, and evaluation process of DICC. They might further aid in understanding the barriers encountered in interprofessional use of DICC and identifying solutions. Furthermore, the results might help to advance new forms of interprofessional communication and to establish collaborations among different stakeholders in the health care setting. This might support a transition toward more coordinated care and help create digital frameworks for integrated patient care. Furthermore, the results might inform stakeholders from different backgrounds, including health care providers, health authorities and management staff, as well as entities developing DICCs.

### Limitations

Our results will need to be interpreted with caution, as our search is limited in terms of language and publication time, which may have led to the exclusion of relevant literature. However, we deem the inclusion of manuscripts written in one of five languages to be quite extensive. In addition, we will not include any grey literature, as we want to address the current gaps in scientific literature. However, including non-peer-reviewed interventions and experiences assessing DICCs may provide further insights.

### Conclusions

To the best of our knowledge, this is the first scoping review synthesizing definitions and dimensions of DICC among health care professionals as described in peer-reviewed studies. Closing this definition gap will provide a differentiated definition system, informing future research activities and implementation projects alike. We additionally expect to identify areas that merit further research in the form of primary or review studies.

## Appendix

Multimedia Appendix 1 Supplementary material.

## Abbreviations

DICC: digitally supported interprofessional communication and collaboration
ICC: interprofessional communication and collaboration
NASSS: nonadoption, abandonment, scale-up, spread, and sustainability
PRISMA-ScR: Preferred Reporting Items for Systematic Reviews and Meta-Analyses extension for Scoping Reviews

## Data Availability

All data supporting the results of the scoping review will be reported in the manuscript, and the entire raw data sets that support the findings will be made available by the authors.
