# Supplementary material for: Conceptualizing Interprofessional Digital Communication and Collaboration in Health Care: Protocol for a Scoping Review
Source: JMIR Res Protoc. 2023 Jun 26;12:e45179. doi: 10.2196/45179 (PMC10337332; doi:10.2196/45179)
Supplement: Multimedia Appendix 1 [file resprot_v12i1e45179_app1.docx]

**Supplementary Material**

Table S1: Search strategy in detail for PubMed search

| Participants, concept, context (PCC scheme) | # | Connector | Search String | Hits (11.12.2022) |
| --- | --- | --- | --- | --- |
|  |  |  |  |  |
| **Communication and collaboration between different healthcare provider grou**ps | 1 |  | trans-disciplin*[Title/Abstract] | 297 |
|  | 2 |  | transdisciplin*[Title/Abstract] | 2,999 |
|  | 3 |  | cross-disciplinar*[Title/Abstract] | 2,216 |
|  | 4 |  | crossdisciplinar*[Title/Abstract] | 2,119 |
|  | 5 |  | inter-disciplin*[Title/Abstract] | 920 |
|  | 6 |  | interdisciplin*[Title/Abstract] | 47,892 |
|  | 7 |  | multi-disciplin*[Title/Abstract] | 8,892 |
|  | 8 |  | multidisciplin*[Title/Abstract] | 119,632 |
|  | 9 |  | multi-profession*[Title/Abstract] | 1,588 |
|  | 10 |  | multiprofession*[Title/Abstract] | 3,234 |
|  | 11 |  | inter-profession*[Title/Abstract] | 1,879 |
|  | 12 |  | interprofession*[Title/Abstract] | 16,172 |
|  | 13 | #1-#12 with OR | (((((((((((trans-disciplin*[Title/Abstract]) OR (transdisciplin*[Title/Abstract])) OR (cross-disciplinar*[Title/Abstract])) OR (crossdisciplinar*[Title/Abstract])) OR (inter-disciplin*[Title/Abstract])) OR (interdisciplin*[Title/Abstract])) OR (multi-disciplin*[Title/Abstract])) OR (multidisciplin*[Title/Abstract])) OR (multi-profession*[Title/Abstract])) OR (multiprofession*[Title/Abstract])) OR (inter-profession*[Title/Abstract])) OR (interprofession*[Title/Abstract]) | 185,543 |
|  | 14 |  | "knowledge transfer"[Title/Abstract] | 2,591 |
|  | 15 |  | information*[Title/Abstract] | 1,489,287 |
|  | 16 |  | Health Information Exchange[MeSH Terms] | 1,080 |
|  | 17 |  | cooperat*[Title/Abstract] | 171,983 |
|  | 18 |  | co-operat*[Title/Abstract] | 12,063 |
|  | 19 |  | collaborat*[Title/Abstract] | 188,735 |
|  | 20 |  | communicat*[Title/Abstract] | 378,139 |
|  | 21 | #14-#20 with OR | (((((("knowledge transfer"[Title/Abstract]) OR (information*[Title/Abstract])) OR (Health Information Exchange[MeSH Terms])) OR (cooperat*[Title/Abstract])) OR (co-operat*[Title/Abstract])) OR (collaborat*[Title/Abstract])) OR (communicat*[Title/Abstract]) | 2,089,524 |
|  | 22 | #13 AND #21 | ((((((((((((trans-disciplin*[Title/Abstract]) OR (transdisciplin*[Title/Abstract])) OR (cross-disciplinar*[Title/Abstract])) OR (crossdisciplinar*[Title/Abstract])) OR (inter-disciplin*[Title/Abstract])) OR (interdisciplin*[Title/Abstract])) OR (multi-disciplin*[Title/Abstract])) OR (multidisciplin*[Title/Abstract])) OR (multi-profession*[Title/Abstract])) OR (multiprofession*[Title/Abstract])) OR (inter-profession*[Title/Abstract])) OR (interprofession*[Title/Abstract])) AND ((((((("knowledge transfer"[Title/Abstract]) OR (information*[Title/Abstract])) OR (Health Information Exchange[MeSH Terms])) OR (cooperat*[Title/Abstract])) OR (co-operat*[Title/Abstract])) OR (collaborat*[Title/Abstract])) OR (communicat*[Title/Abstract])) | 52,850 |
|  | 23 |  | "integrated care"[Title/Abstract] | 6,670 |
|  | 24 |  | Intersectoral Collaboration[MeSH Terms] | 2,567 |
|  | 25 |  | Interdisciplinary Communication[MeSH Terms] | 18,111 |
|  | 26 | #23 OR #24 OR #25 | (("integrated care"[Title/Abstract]) OR (Intersectoral Collaboration[MeSH Terms])) OR (Interdisciplinary Communication[MeSH Terms]) | 26,343 |
|  | 27 | #22 OR #26 | (((((((((((((trans-disciplin*[Title/Abstract]) OR (transdisciplin*[Title/Abstract])) OR (cross-disciplinar*[Title/Abstract])) OR (crossdisciplinar*[Title/Abstract])) OR (inter-disciplin*[Title/Abstract])) OR (interdisciplin*[Title/Abstract])) OR (multi-disciplin*[Title/Abstract])) OR (multidisciplin*[Title/Abstract])) OR (multi-profession*[Title/Abstract])) OR (multiprofession*[Title/Abstract])) OR (inter-profession*[Title/Abstract])) OR (interprofession*[Title/Abstract])) AND ((((((("knowledge transfer"[Title/Abstract]) OR (information*[Title/Abstract])) OR (Health Information Exchange[MeSH Terms])) OR (cooperat*[Title/Abstract])) OR (co-operat*[Title/Abstract])) OR (collaborat*[Title/Abstract])) OR (communicat*[Title/Abstract]))) OR ((("integrated care"[Title/Abstract]) OR (Intersectoral Collaboration[MeSH Terms])) OR (Interdisciplinary Communication[MeSH Terms])) | 75,493 |
| Digital tools | 28 |  | Health Information Systems[MeSH Terms] | 1,587 |
|  | 29 |  | Ambulatory Care Information Systems[MeSH Terms] | 1,171 |
|  | 30 |  | Information Technology[MeSH Terms] | 733 |
|  | 31 |  | technolog*[Title/Abstract] | 626,873 |
|  | 32 |  | socio-techni*[Title/Abstract] | 745 |
|  | 33 |  | sociotechni*[Title/Abstract] | 1,547 |
|  | 34 |  | mHealth[Title/Abstract] | 8,867 |
|  | 35 |  | eHealth[Title/Abstract] | 8,982 |
|  | 36 |  | digit*[Title/Abstract] | 252,283 |
|  | 37 |  | Electronic Health Records[MeSH Terms] | 26,794 |
|  | 38 |  | Public Health Informatics[MeSH Terms] | 1,250 |
|  | 39 |  | messag*[Title/Abstract] | 71,943 |
|  | 40 |  | messeng*[Title/Abstract] | 82,691 |
|  | 41 |  | app[Title/Abstract] | 37,310 |
|  | 42 |  | video*[Title/Abstract] | 160,804 |
|  | 43 |  | phone[Title/Abstract] | 32,395 |
|  | 44 |  | E-Mail*[Title/Abstract] | 9,648 |
|  | 45 |  | "E Mail"[Title/Abstract] | 7,910 |
|  | 46 |  | "E Mails"[Title/Abstract] | 895 |
|  | 47 |  | Email*[Title/Abstract] | 21,852 |
|  | 48 |  | "electronic mail"[Title/Abstract] | 823 |
|  | 49 |  | "electronic mails"[Title/Abstract] | 16 |
|  | 50 |  | "social media"[Title/Abstract] | 25,573 |
|  | 51 |  | WhatsApp[Title/Abstract] | 1,436 |
|  | 52 |  | Facebook[Title/Abstract] | 6,031 |
|  | 53 |  | Viber[Title/Abstract] | 31 |
|  | 54 |  | WeChat[Title/Abstract] | 948 |
|  | 55 |  | Telegram[Title/Abstract] | 168 |
|  | 56 |  | Kakaotalk[Title/Abstract] | 5 |
|  | 57 | #28-#56 with OR | ((((((((((((((((((((((((((((Health Information Systems[MeSH Terms]) OR (Ambulatory Care Information Systems[MeSH Terms])) OR (Information Technology[MeSH Terms])) OR (technolog*[Title/Abstract])) OR (socio-techni*[Title/Abstract])) OR (sociotechni*[Title/Abstract])) OR (mHealth[Title/Abstract])) OR (eHealth[Title/Abstract])) OR (digit*[Title/Abstract])) OR (Electronic Health Records[MeSH Terms])) OR (Public Health Informatics[MeSH Terms])) OR (messag*[Title/Abstract]))  OR (messeng*[Title/Abstract])) OR (app[Title/Abstract])) OR (video*[Title/Abstract])) OR (phone[Title/Abstract])) OR (E-Mail*[Title/Abstract])) OR (Email*[Title/Abstract])) OR ("E Mail"[Title/Abstract])) OR ("E Mails"[Title/Abstract])) OR ("Electronic Mail"[Title/Abstract])) OR ("Electronic Mails"[Title/Abstract])) OR ("social media"[Title/Abstract])) OR (WhatsApp[Title/Abstract])) OR (Facebook[Title/Abstract])) OR (Viber[Title/Abstract])) OR (WeChat[Title/Abstract])) OR (Telegram[Title/Abstract]))  OR (Kakaotalk[Title/Abstract]) | 1,254,069 |
| Healthcare setting | 58 |  | health*[Title/Abstract] | 3,478,328 |
|  | 59 |  | hospital*[Title/Abstract] | 1,566,327 |
|  | 60 |  | care*[Title/Abstract] | 2,049,851 |
|  | 61 |  | caring[Title/Abstract] | 49,262 |
|  | 62 | #58-#61 with OR | (((health*[Title/Abstract]) OR (hospital*[Title/Abstract])) OR (care*[Title/Abstract])) OR (caring[Title/Abstract]) | 5,596,504 |
| Total | 63 | #27 AND #57 AND #62 | (((((((((((((((trans-disciplin*[Title/Abstract]) OR (transdisciplin*[Title/Abstract])) OR (cross-disciplinar*[Title/Abstract])) OR (crossdisciplinar*[Title/Abstract])) OR (inter-disciplin*[Title/Abstract])) OR (interdisciplin*[Title/Abstract])) OR (multi-disciplin*[Title/Abstract])) OR (multidisciplin*[Title/Abstract])) OR (multi-profession*[Title/Abstract])) OR (multiprofession*[Title/Abstract])) OR (inter-profession*[Title/Abstract])) OR (interprofession*[Title/Abstract])) AND ((((((("knowledge transfer"[Title/Abstract]) OR (information*[Title/Abstract])) OR (Health Information Exchange[MeSH Terms])) OR (cooperat*[Title/Abstract])) OR (co-operat*[Title/Abstract])) OR (collaborat*[Title/Abstract])) OR (communicat*[Title/Abstract]))) OR ((("integrated care"[Title/Abstract]) OR (Intersectoral Collaboration[MeSH Terms])) OR (Interdisciplinary Communication[MeSH Terms]))) AND (((((((((((((((((((((((((((((Health Information Systems[MeSH Terms]) OR (Ambulatory Care Information Systems[MeSH Terms])) OR (Information Technology[MeSH Terms])) OR (technolog*[Title/Abstract])) OR (socio-techni*[Title/Abstract])) OR (sociotechni*[Title/Abstract])) OR (mHealth[Title/Abstract])) OR (eHealth[Title/Abstract])) OR (digit*[Title/Abstract])) OR (Electronic Health Records[MeSH Terms])) OR (Public Health Informatics[MeSH Terms])) OR (messag*[Title/Abstract])) OR (messeng*[Title/Abstract])) OR (app[Title/Abstract])) OR (video*[Title/Abstract])) OR (phone[Title/Abstract])) OR (E-Mail*[Title/Abstract])) OR ("E Mail"[Title/Abstract])) OR ("E Mails"[Title/Abstract])) OR (Email*[Title/Abstract])) OR ("electronic mail"[Title/Abstract])) OR ("electronic mails"[Title/Abstract])) OR ("social media"[Title/Abstract])) OR (WhatsApp[Title/Abstract])) OR (Facebook[Title/Abstract])) OR (Viber[Title/Abstract])) OR (WeChat[Title/Abstract])) OR (Telegram[Title/Abstract])) OR (Kakaotalk[Title/Abstract]))) AND ((((health*[Title/Abstract]) OR (hospital*[Title/Abstract])) OR (care*[Title/Abstract])) OR (caring[Title/Abstract])) | 7,261 |
|  | 64 |  | Filters: from 2012 onwards, English, German, French, Portuguese, Spanish | 5,694 |

Table S2: Search strategy for individual databases

| Database | Search String | Filters used | Hits with filters |
| --- | --- | --- | --- |
|  |  |  |  |
| **CINAHL** | (((MH (intersectoral collaboration) OR TI ( "integrated care" OR trans-disciplin* OR transdisciplin* OR cross-disciplinar* OR crossdisciplinar* OR inter-disciplin* OR interdisciplin* OR multi-disciplin* OR multidisciplin* OR multiprofession* OR multi-profession* OR inter-profession* OR interprofession* ) OR AB ( "integrated care" OR trans-disciplin* OR transdisciplin* OR cross-disciplinar* OR crossdisciplinar* OR inter-disciplin* OR interdisciplin* OR multi-disciplin* OR multidisciplin* OR multiprofession* OR multi-profession* OR inter-profession* OR interprofession* ))) AND (((MH (health information exchange) OR TI ( "knowledge transfer" OR information* OR cooperat* OR co-operat* OR collaborat* OR communicat* ) OR AB ( "knowledge transfer" OR information* OR cooperat* OR co-operat* OR collaborat* OR communicat* )))  AND (((MH (health information systems OR ambulatory care information systems OR information technology OR electronic health records OR public health informatics) OR TI ( technolog* OR socio-techni* OR sociotechni* OR mHealth OR eHealth OR digit* OR messag* OR messeng* OR app OR video* OR phone OR email OR emails OR "electronic mail" OR "electronic mails" OR "social media" OR whatsapp OR facebook OR viber OR wechat OR OR telegram OR Kakaotalk ) OR AB ( technolog* OR socio-techni* OR sociotechni* OR mHealth OR eHealth OR digit* OR messag* OR messeng* OR app OR video* OR phone OR email OR emails OR "electronic mail" OR "electronic mails" OR "social media" OR whatsapp OR facebook OR viber OR wechat OR OR telegram OR Kakaotalk ))) AND (TI ( health OR hospital* OR care* OR caring ) OR AB ( health OR hospital* OR care* OR caring ) ) | Language: English, German, French, Portuguese, Spanish    Publication year 2012-2023 | 2,676 |
| **Embase** | (((((&apos;integrated care&apos;:ti,ab) OR (&apos;Intersectoral Collaboration&apos;:ti,ab)) OR (&apos;Interdisciplinary Communication&apos;:ti,ab)) OR (((((((((((((trans-disciplin*:ti,ab) OR (transdisciplin*:ti,ab)) OR (cross-disciplinar*:ti,ab)) OR (crossdisciplinar*:ti,ab)) OR (inter-disciplin*:ti,ab)) OR (interdisciplin*:ti,ab)) OR (multi-disciplin*:ti,ab)) OR (multidisciplin*:ti,ab)) OR (multi-profession*:ti,ab)) OR (multiprofession*:ti,ab)) OR (inter-profession*:ti,ab)) OR (interprofession*:ti,ab)) AND (((((((&apos;knowledge transfer&apos;:ti,ab) OR (information*:ti,ab)) OR (&apos;Health Information Exchange&apos;:ti,ab)) OR (cooperat*:ti,ab)) OR (co-operat*:ti,ab)) OR (collaborat*:ti,ab)) OR (communicat*:ti,ab)))) AND (((((((((((((((((((((((((((((&apos;Health Information Systems&apos;:ti,ab) OR (&apos;Ambulatory Care Information Systems&apos;:ti,ab)) OR (&apos;Information Technology&apos;:ti,ab)) OR (technolog*:ti,ab)) OR (socio-techni*:ti,ab)) OR (sociotechni*:ti,ab)) OR (mHealth:ti,ab)) OR (eHealth:ti,ab)) OR (digit*:ti,ab)) OR (&apos;Electronic Health Records&apos;:ti,ab)) OR (&apos;Public Health Informatics&apos;:ti,ab)) OR (messag*:ti,ab)) OR (messeng*:ti,ab)) OR (app:ti,ab)) OR (video*:ti,ab)) OR (phone:ti,ab)) OR (E-Mail*:ti,ab)) OR (Email*:ti,ab)) OR (&apos;E Mail&apos;:ti,ab)) OR (&apos;E Mails&apos;:ti,ab)) OR (&apos;Electronic Mail&apos;:ti,ab)) OR (&apos;Electronic Mails&apos;:ti,ab)) OR (&apos;social media&apos;:ti,ab)) OR (WhatsApp:ti,ab)) OR (Facebook:ti,ab)) OR (Viber:ti,ab)) OR (WeChat:ti,ab)) OR (Telegram:ti,ab)) OR (Kakaotalk:ti,ab))) AND ((((health*:ti,ab) OR (hospital*:ti,ab)) OR (care*:ti,ab)) OR (caring:ti,ab)) | Language: English, German, French, Portuguese, Spanish    Publication year 2012-2023 | 8,733 |
| **PubMed** | (((((((((((((((trans-disciplin*[Title/Abstract]) OR (transdisciplin*[Title/Abstract])) OR (cross-disciplinar*[Title/Abstract])) OR (crossdisciplinar*[Title/Abstract])) OR (inter-disciplin*[Title/Abstract])) OR (interdisciplin*[Title/Abstract])) OR (multi-disciplin*[Title/Abstract])) OR (multidisciplin*[Title/Abstract])) OR (multi-profession*[Title/Abstract])) OR (multiprofession*[Title/Abstract])) OR (inter-profession*[Title/Abstract])) OR (interprofession*[Title/Abstract])) AND ((((((("knowledge transfer"[Title/Abstract]) OR (information*[Title/Abstract])) OR (Health Information Exchange[MeSH Terms])) OR (cooperat*[Title/Abstract])) OR (co-operat*[Title/Abstract])) OR (collaborat*[Title/Abstract])) OR (communicat*[Title/Abstract]))) OR ((("integrated care"[Title/Abstract]) OR (Intersectoral Collaboration[MeSH Terms])) OR (Interdisciplinary Communication[MeSH Terms]))) AND (((((((((((((((((((((((((((((Health Information Systems[MeSH Terms]) OR (Ambulatory Care Information Systems[MeSH Terms])) OR (Information Technology[MeSH Terms])) OR (technolog*[Title/Abstract])) OR (socio-techni*[Title/Abstract])) OR (sociotechni*[Title/Abstract])) OR (mHealth[Title/Abstract])) OR (eHealth[Title/Abstract])) OR (digit*[Title/Abstract])) OR (Electronic Health Records[MeSH Terms])) OR (Public Health Informatics[MeSH Terms])) OR (messag*[Title/Abstract])) OR (messeng*[Title/Abstract])) OR (app[Title/Abstract])) OR (video*[Title/Abstract])) OR (phone[Title/Abstract])) OR (E-Mail*[Title/Abstract])) OR ("E Mail"[Title/Abstract])) OR ("E Mails"[Title/Abstract])) OR (Email*[Title/Abstract])) OR ("electronic mail"[Title/Abstract])) OR ("electronic mails"[Title/Abstract])) OR ("social media"[Title/Abstract])) OR (WhatsApp[Title/Abstract])) OR (Facebook[Title/Abstract])) OR (Viber[Title/Abstract])) OR (WeChat[Title/Abstract])) OR (Telegram[Title/Abstract])) OR (Kakaotalk[Title/Abstract]))) AND ((((health*[Title/Abstract]) OR (hospital*[Title/Abstract])) OR (care*[Title/Abstract])) OR (caring[Title/Abstract])) | Language: English, German, French, Portuguese, Spanish    Publication year 2012-2023 | 5,694 |
| **PsycInfo** | ((Title:("integrated care") OR Title:("intersectoral collaboration") OR Title:("interdisciplinary communication") OR Title:(trans-disciplin*) OR Title:(transdiciplin*) OR Title:(cross-diciplinar*) OR Title:(crossdisciplinar*) OR Title:(inter-discipplin*) OR Title:(interdisciplin*) OR Title:(multi-disciplin*) OR Title:(multidisciplin*) OR Title:(multi-profession*) OR Title:(multiprofession*) OR Title:(inter-profession) OR Title:(interprofession)) OR (Abstract:("integrated care") OR Abstract:("intersectoral collaboration") OR Abstract:("interdisciplinary communication") OR Abstract:(trans-disciplin*) OR Abstract:(transdiciplin*) OR Abstract:(cross-diciplinar*) OR Abstract:(crossdisciplinar*) OR Abstract:(inter-discipplin*) OR Abstract:(interdisciplin*) OR Abstract:(multi-disciplin*) OR Abstract:(multidisciplin*) OR Abstract:(multi-profession*) OR Abstract:(multiprofession*) OR Abstract:(inter-profession) OR Abstract:(interprofession))) AND ((Title:(health*) OR Title:(hospital*) OR Title:(care*) OR Title:(caring)) OR (Abstract:(health*) OR Abstract:(hospital*) OR Abstract:(care*) OR Abstract:(caring))) AND ((Title:("Health Information Systems") OR Title:("ambulatory care information systems") OR Title:(technolog*) OR Title:(socio-techni*) OR Title:(sociotechni*) OR Title:(mHealth) OR Title:(eHealth) OR Title:(digit*) OR Title:("electronic health records") OR Title:("public health informatics") OR Title:(messag*) OR Title:(messeng*) OR Title:(app) OR Title:(video*) OR Title:(phone) OR Title:(e-mail) OR Title:(email) OR Title:(e-mails) OR Title:(emails) OR Title:("electronic mail") OR Title:("electronic mails") OR Title:("social media") OR Title:(whatsapp) OR Title:(facebook) OR Title:(Viber) OR Title:(wechat) OR Title:(Telegram) OR Title:(Kakaotalk)) OR (Abstract:("Health Information Systems") OR Abstract:("ambulatory care information systems") OR Abstract:(technolog*) OR Abstract:(socio-techni*) OR Abstract:(sociotechni*) OR Abstract:(mHealth) OR Abstract:(eHealth) OR Abstract:(digit*) OR Abstract:("electronic health records") OR Abstract:("public health informatics") OR Abstract:(messag*) OR Abstract:(messeng*) OR Abstract:(app) OR Abstract:(video*) OR Abstract:(phone) OR Abstract:(e-mail) OR Abstract:(email) OR Abstract:(e-mails) OR Abstract:(emails) OR Abstract:("electronic mail") OR Abstract:("electronic mails") OR Abstract:("social media") OR Abstract:(whatsapp) OR Abstract:(facebook) OR Abstract:(Viber) OR Abstract:(wechat) OR Abstract:(Telegram) OR Abstract:(Kakaotalk))) | Language: English, German, French, Portuguese, Spanish    Publication year 2012-2023 | 1,617 |
| **SCOPUS** | (((((TITLE-ABS("integrated care")) OR (TITLE-ABS-KEY("Intersectoral Collaboration"))) OR (TITLE-ABS-KEY("Interdisciplinary Communication"))) OR (((((((((((((TITLE-ABS(trans-disciplin*)) OR (TITLE-ABS(transdisciplin*))) OR (TITLE-ABS(cross-disciplinar*))) OR (TITLE-ABS(crossdisciplinar*))) OR (TITLE-ABS(inter-disciplin*))) OR (TITLE-ABS(interdisciplin*))) OR (TITLE-ABS(multi-disciplin*))) OR (TITLE-ABS(multidisciplin*))) OR (TITLE-ABS(multi-profession*))) OR (TITLE-ABS(multiprofession*))) OR (TITLE-ABS(inter-profession*))) OR (TITLE-ABS(interprofession*))) AND (((((((TITLE-ABS("knowledge transfer")) OR (TITLE-ABS(information*))) OR (TITLE-ABS-KEY("Health Information Exchange"))) OR (TITLE-ABS(cooperat*))) OR (TITLE-ABS(co-operat*))) OR (TITLE-ABS(collaborat*))) OR (TITLE-ABS(communicat*))))) AND (((((((((((((((((((((((((((((TITLE-ABS-KEY("Health Information Systems")) OR (TITLE-ABS-KEY("Ambulatory Care Information Systems"))) OR (TITLE-ABS-KEY("Information Technology"))) OR (TITLE-ABS(technolog*))) OR (TITLE-ABS(socio-techni*))) OR (TITLE-ABS(sociotechni*))) OR (TITLE-ABS(mHealth))) OR (TITLE-ABS(eHealth))) OR (TITLE-ABS(digit*))) OR (TITLE-ABS-KEY("Electronic Health Records"))) OR (TITLE-ABS-KEY("Public Health Informatics"))) OR (TITLE-ABS(messag*))) OR (TITLE-ABS(messeng*))) OR (TITLE-ABS(app))) OR (TITLE-ABS(video*))) OR (TITLE-ABS(phone))) OR (TITLE-ABS(E-Mail*))) OR (TITLE-ABS(Email*))) OR (TITLE-ABS("E Mail"))) OR (TITLE-ABS("E Mails"))) OR (TITLE-ABS("Electronic Mail"))) OR (TITLE-ABS("Electronic Mails"))) OR (TITLE-ABS("social media"))) OR (TITLE-ABS(WhatsApp))) OR (TITLE-ABS(Facebook))) OR (TITLE-ABS(Viber))) OR (TITLE-ABS(WeChat))) OR (TITLE-ABS(Telegram))) OR (TITLE-ABS(Kakaotalk)))) AND ((((TITLE-ABS(health*)) OR (TITLE-ABS(hospital*))) OR (TITLE-ABS(care*))) OR (TITLE-ABS(caring))) | Language: English, German, French, Portuguese, Spanish    Publication year 2012-2023 | 8,090 |
